# Dataset: Knowledge and attitude retention following an implicit bias classroom workshop

**DOI:** 10.12688/f1000research.74442.1

**Published:** 2022-01-11

**Authors:** Yuanyuan Zhou, Joel Purkiss, Malvika Juneja, Jocelyn Greely, Anitra Beasley, Anne Gill

**Affiliations:** 1 Division of Evaluation, Assessment, and Education Research, Baylor College of Medicine, One Baylor Plaza, Houston, Texas, 77030, USA; 2 Department of Education, Innovation, and Technology, Baylor College of Medicine, One Baylor Plaza, Houston, Texas, 77030, USA; 3 Department of Family and Community Medicine, Baylor College of Medicine, One Baylor Plaza, Houston, Texas, 77030, USA; 4 Department of Obstetrics & Gynecology, Baylor College of Medicine, One Baylor Plaza, Houston, Texas, 77030, USA; 5 Department of Pediatrics, Baylor College of Medicine, One Baylor Plaza, Houston, Texas, 77030, USA

**Keywords:** implicit bias, classroom workshop, repeated measures, Likert scales survey, unconscious bias

## Abstract

**Background**: Baylor College of Medicine provides a classroom-based implicit bias workshop to all third-year medical students to increase students’ awareness of their unconscious bias and develop strategies for reducing health care disparities. The workshop meets our immediate goals and objectives. However, we are unsure if the benefit would be long-term or diminish over time.

**Methods**: To examine the concept retention from the implicit bias classroom workshop, we administered a self-developed seven-item seven-point Likert-scale survey to our medical students at pre-, post-, and one-year post-workshop attendance.

**Results**: The data set was comprised of survey results from two cohorts of our third and fourth-year medical students from 2018 to 2020 and included 289 completed records at three measurement points. The data included: Student Identifiers, Sex, Race/Ethnicity, Student Enrollment Type, Cohort, and three repeated measures results for each of the seven items, which were documented in wide format. The data may be of interest to those who wish to examine how factors including elapsed time, race, and sex may associate with attitudes and understandings of implicit bias following related training, and those interested in analytical methods on longitudinal research in general.

## Introduction

**Figure 1.**
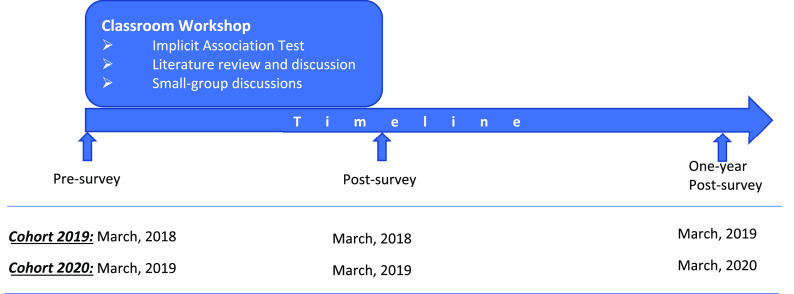
Implicit bias workshop schematic overview and timeline.

Baylor College of Medicine (BCM) has a long history of educating medical students to develop awareness of their potential inherent bias towards specific racial/ethnic groups that might influence their medical decision-making. ^1^^–^^4^ Educators and researchers at BCM developed a classroom-based implicit bias workshop, which has been part of the third-year medical student curriculum since 2008. ^5^ The workshop is currently part of a third-year course on social determinants of health and includes administration of the Implicit Association Test ^6^ (IAT), student review of two articles ^7^^,^^8^ about implicit bias in medical education, and small-group discussions. The IAT was used to examine students’ inherent predispositions based on certain demographic groups (e.g., race, weight), with the aim of triggering students’ self-awareness and self-reflection of their unconscious bias. The students were told to read the two articles before the session and to discuss them during the small group session. The literature review allowed examination of how such unconscious bias may influence physicians’ clinical decision-making and was followed by active small-group discussion which fostered engagement with management strategies for reducing health care disparities (a schematic overview of the research design can be found in Figure 1).

Such educational practices have demonstrated an effective immediate influence on students’ awareness and management of implicit bias. ^4^ However, we were uncertain whether this immediate impact following the one-session workshop would be sustained over time. If the educational effects of the IAT workshop diminish over time, a one-time classroom workshop may not be sufficient in preventing health care disparities among certain social groups. Offering more training in this area to our medical students would require additional time and resources in an already compressed curriculum. A better understanding of how the effects of implicit bias training persist following a classroom workshop would enable medical schools to design and develop curricula which maximize training efficiency and students’ future development as physicians.

The research manuscript generated from this study has been Accepted by Medical Teacher for publication and is in press. However, the data might be valuable for those wishing to pursue additional secondary analyses of related research topics, or a meta-analysis that combines multiple independent scientific studies of similar topics. These data might also be valuable for those interested in using other analytical methods to investigate the same research question or even for new longitudinal research simply focusing on the analytical methods in general, beyond the topic of the implicit bias.

## Methods

### Study design

This data set was collected as part of ongoing curriculum evaluation and improvement process. However, when aggregated together for the purposes of this project, the data comprise a longitudinal observational study that measures students’ attitudinal change over three repeated measures: immediately before participating in the workshop, immediately after participating in the workshop, and one year after participating in the workshop (measurement timing can be found in Figure 1).

The implicit bias workshop was administered within the required Determinants, Disparities, and Social Health of Populations (D-DASH) course. All third-year students who took the D-DASH course were considered as potential participants in this study. They were informed of the purpose of this study prior to agreeing to complete the survey and learned that information collected through the survey would be used as part of a research protocol. They received the pre-and post-surveys through the web-based course evaluation platform e*Value.

Approximately one year later, we conducted a one-year post-survey of the students during their fourth year as part of their required capstone course, Advanced Physician Experience (APEX). All students in APEX courses were informed again that the survey was part of a research protocol and they were voluntarily agreeing to participate the study.

### Study population

The study was based on data collected between 2018 and 2020 from two cohorts of medical students (Class of 2019: n = 168; and Class of 2020: n = 185). Each class of students first received the pre- and post-surveys in their third year (MS3) spring D-DASH course and then completed the same survey the following spring in their fourth year (MS4). Due to merged curricular tracks, there was a small portion of students who did not receive the implicit bias surveys and training in their MS3 spring, but still received the one-year post-workshop-survey in their MS4 spring term. These off-track students did not meet the research criterion and were excluded from the data analysis.

### Data collection

Members of the BCM research team created the survey for this study in 2008, when the implicit bias workshop was offered to students and it has been revised and refined several times based on piloted data and students’ feedback. The final version included seven questions evaluating students’ thoughts on questions regarding a physician’s or individual’s perceptions and management of unconscious bias. The survey was in a Likert-style format, with seven options ranging from strongly disagree (1) to strongly agree (7) (the survey can be found in the *extended data* ^9^).

Surveys were distributed through the course evaluation platform e*Value and were active for two weeks to allow students to complete the surveys. The response rates ranged from 92.2% to 100.0% (see Table 1 for complete response rates). Survey data were exported as Excel data files and were saved in a secure/encrypted web portal within the BCM Medicine network. The original data were identifiable, but we deidentified data for subsequent analysis.

**Table 1.** Response rate for each data-collection period, by cohort.

Cohort	Completed during D-DASH course		Completed during APEX course
	Pre-workshop survey	Post-workshop survey	One-year post-workshop survey
Class of 2019	166 ^a^/168 ^b^ 98.8%	162 ^a^/168 ^b^ 96.4%	155 ^a^/167 ^b^ 92.8%
Class of 2020	185 ^a^/185 ^b^ 100.0%	184 ^a^/185 ^b^ 99.5%	177 ^a^/192 ^b^ 92.2%

^a^ Number of students completing the survey.

^b^ Number of students receiving the survey.

The data file included 26 variables: Student Identifier, Gender, Race/Ethnicity, Student Enrollment Type, Cohort, and 21 variables for the survey results. Student Identifier is a unique number for each student. We used randomly generated 10-digit numbers to replace the original school identification numbers to promote confidentiality of respondent data. Gender was self-defined by students. We allowed students to identify a gender other than male or female with an ‘other’ option and space to write their own answer, but all students identified themselves as either male or female. Students also selected their race/ethnicity. We merged ethnicities into more general and commonly used categories and were left with four options: White, Black or African American, Hispanic, Asian, other Asian, or ‘prefer not to answer’. Student Enrollment Type was a dichotomous variable (“0” or “1”) used to differentiate those who had regular curriculum scheduling for the period under study (“0”) and those who had an irregular curriculum schedule (“1”). There were 17 students in the latter category who completed the one-year post-survey two years after the workshop. These 17 students did not meet the criterion for inclusion in the primary analysis. However, this small sub-sample provided a less robust but still intriguing opportunity to examine for retention of the implicit bias training following an even longer elapsed period after the classroom workshop had been conducted.

Cohort included the 2019 Cohort who took the pre- and post-survey in 2018, and the one-year post-survey in 2019; and Cohort 2020 who took the pre- and post-survey in 2019, and the one-year post-survey in 2020. The survey results have 21 variables because the survey had seven Likert-style items, and each was measured three times. The Likert-style items were coded in numerical format, ranging from 1 to 7 (1 for Strongly Disagree, 7 for Strongly Agree).

We merged the three longitudinal surveys’ results into one file using the unique identifiers assigned to each student. The dataset can be found in *underlying data*. ^9^ We deleted 94 records that did not meet the research criterion: 37 records for those without the pre-survey, three records for those without the post-survey, and 54 records for those without the one-year post-survey. Those without the pre-or one-year post-survey were likely to have irregular curriculum scheduling due to a leave of absence or enrollment in an extended-time and off-cycle dual degree program. Those without the post-survey likely failed to submit responses to the post-survey.

A total of 289 records were available with completed measures at three measurement points, of which 272 students were from the regular curriculum tracks (130 from the Class of 2019, and 142 from the Class of 2020), with the remaining 17 having irregular curriculum scheduling (see Table 2). Resulting data were then merged with race/ethnicity, and gender data self-identified by students which was collected previously during the medical school admissions process. Students were aware that the study may report demographics to describe the sample composition.

**Table 2.** Measurement timing for each cohort and valid sample collected.

Cohort	Measurement timing			Valid sample
	Pre	Post	One-year post	
Class of 2019	2018	2018	2019	n = 130
	2018	2018	2020	n = 17 ^*^
Class of 2020	2019	2019	2020	n = 142

^*^ These 17 students on irregular curriculum schedules actually completed the “One-Year Post” survey two years after the workshop.

## Data validation

The data set has been proofread for human errors. A frequency summary table ( Table 3) was created using the Pivot function in Microsoft Excel. Measurement results were negatively skewed to more positive options but following application of sampling criteria. There were no missing data for any of the three repeated measures. We evaluated the survey quality using this dataset. Cronbach’s alpha measures the internal consistency of survey items, and ranged from 0.86 to 0.87 between the pre-, post-, and one-year post surveys. The seven items had moderate to high inter-item correlations and had consistent patterns across the pre-, post-, and one-year post surveys. The first factor extracted by Exploratory Factor Analysis explains more than half of the variances, which provided primary evidence of a unidimensional structure of the survey. The results provided some evidence that the survey was still solid and reliable. More detailed results for survey quality can be found in Table 4.

**Table 3.** Frequency of choices for each survey question at three measurement time-points.

Survey questions	Measurement timing	Frequency							
		Strongly disagree	Moderately disagree	Slightly disagree	Neither agree nor disagree	Slightly agree	Moderately agree	Strongly agree	Total
Q1	Pre	8	1	5	9	24	97	145	289
	Post	4	4	1	3	20	82	175	289
	One-year post	4	1	1	9	16	88	170	289
Q2	Pre	2	2	1	10	26	79	169	289
	Post		3		6	16	71	193	289
	One-year post			2	8	18	67	194	289
Q3	Pre	2	2		10	19	80	176	289
	Post	1	1		5	16	71	195	289
	One-year post				10	13	55	211	289
Q4	Pre	2	4	6	13	56	91	117	289
	Post	4	5	5	8	35	86	146	289
	One-year post		5	2	14	42	104	122	289
Q5	Pre	2	3	4	16	29	86	149	289
	Post	1	2	2	6	20	80	178	289
	One-year post		1	6	10	17	78	177	289
Q6	Pre		3	7	18	51	106	104	289
	Post		1	2	8	36	107	135	289
	One-year post		1	3	20	26	112	127	289
Q7	Pre		5	8	32	60	113	71	289
	Post		1	2	13	53	109	111	289
	One-year post		1	4	16	51	120	97	289

**Table 4.** Specification of the survey.

	Cronbach’s alpha	% of first factor variance	Inter-item correlation coefficient
Pre survey	0.86	57%	0.19-0.89
Post survey	0.86	59%	0.20-0.90
One-year post survey	0.87	59%	0.16-0.90

## Ethics statement

This study and the publication of the data set was approved by The Institutional Review Board for Human Subject Research at BCM (protocol number: H-45073). Written documentation of consent was waived for this study by the review board due to its low-risk nature. The participants were informed of the purpose of the survey while they took the D-DASH and APEX course and that taking the survey was optional, and non-participation would not impact their course grade. They were informed that, by taking the survey, they were voluntarily agreeing to participate in the study. Students were aware that all survey data would be kept confidential, and data would be deidentified for analysis and reporting.

## Data availability

### Underlying data

DANS: Implicit bias classroom workshop data. https://doi.org/10.17026/dans-zrv-mxsm. ^9^

This project contains the following underlying data:

- DataFileImplicitBias.xlsx (dataset and code book for the variables)

### Extended data

This project contains the following extended data

- Implicit bias survey.pdf (copy of survey).

Data are available under the terms of the Creative Commons Zero “No rights reserved” data waiver (CC0 1.0 Public domain dedication).
