# Supplementary material for: Dataset: Knowledge and attitude retention following an implicit bias classroom workshop
Source: F1000Res. 2022 Jan 11;11:25. [Version 1] doi: 10.12688/f1000research.74442.1 (PMC8874035; doi:10.12688/f1000research.74442.1)
Supplement: Peer review history

# Peer review: Reviewer response for version 1

**DOI:** 10.5256/f1000research.78200.r123367

**Published:** 2022-03-11

**Authors:** Courtney West

**Affiliations:** 1 College of Osteopathic Medicine, Sam Houston State University, Conroe, TX, USA

The rationale for creating the dataset is clearly described. The purpose was to “examine concept retention” from an implicit bias classroom workshop using a seven-item seven-point Likert-scale survey. “The data set was comprised of survey results from two cohorts of third and fourth-year medical students from 2018 to 2020 and included 289 completed records at three measurement points.”

 The manuscript is well written, and the description of the protocols are appropriate and sound. The results are clearly presented in the text and in the attached tables. They are consistent with the methods used. 

 The authors provide sufficient details of how they examined concept retention which would allow replication by others. The data collection methods have been outlined effectively and the data validation process is clearly described so that another researcher could replicate and extend the study. This data note highlights a robust approach that may be adopted and utilized to examine knowledge and attitude retention over time.

 The datasets are presented in a useable and accessible format. For example, the authors provided a data dictionary/code book on the second tab of the excel data file. In addition, a pdf document including the implicit bias workshop survey was also included as extended data.

Are sufficient details of methods and materials provided to allow replication by others?

Yes

Is the rationale for creating the dataset(s) clearly described?

Yes

Are the datasets clearly presented in a useable and accessible format?

Yes

Are the protocols appropriate and is the work technically sound?

Yes

Reviewer Expertise:

My research interests include, but are not limited to, learning and study strategies, instructional techniques, program evaluation, professional development, and team based collaborative care.

I confirm that I have read this submission and believe that I have an appropriate level of expertise to confirm that it is of an acceptable scientific standard.

# Peer review: Reviewer response for version 1

**DOI:** 10.5256/f1000research.78200.r122372

**Published:** 2022-02-24

**Authors:** Ling Ning

**Affiliations:** 1 Office of Assessment and Planning, University of Colorado Boulder, Boulder, CO, USA

Thank you for the opportunity to review the manuscript ***Dataset: Knowledge and attitude retention following an implicit bias classroom workshop for F1000Research***. The article was very well written with clear and precise language. The authors did a very good job in articulating the purposes of making the data available for public access. The authors also illustrate very clearly the study design, study population, data collection, aggregation, and validation process.

 Minor points:

 Page 4 (of the pdf version of the article): about ethnicities: “ …were left with four options…”, not very clear. Not exactly four options.

Are sufficient details of methods and materials provided to allow replication by others?

Yes

Is the rationale for creating the dataset(s) clearly described?

Yes

Are the datasets clearly presented in a useable and accessible format?

Yes

Are the protocols appropriate and is the work technically sound?

Yes

Reviewer Expertise:

My research focuses on multilevel/longitudinal modeling of data with complex structures, propensity score analysis, and value-added assessment.

I confirm that I have read this submission and believe that I have an appropriate level of expertise to confirm that it is of an acceptable scientific standard.

# Peer review: Reviewer response for version 1

**DOI:** 10.5256/f1000research.78200.r119372

**Published:** 2022-02-04

**Authors:** Xuejun Ji

**Affiliations:** 1 Department of Educational and Counselling Psychology, and Special Education, University of British Columbia (UBC), Vancouver, BC, Canada

The rationale for the dataset building is fully articulated. The users can access the dataset directly and replicate the results easily. No missing data were found. The codebook is available for users, which allows users to conduct data wrangling and further analysis. Overall, the submitted dataset is a good source for any further studies and replication studies.

Are sufficient details of methods and materials provided to allow replication by others?

Yes

Is the rationale for creating the dataset(s) clearly described?

Yes

Are the datasets clearly presented in a useable and accessible format?

Yes

Are the protocols appropriate and is the work technically sound?

Yes

Reviewer Expertise:

Psychometrics, OSCE examine, validity and validation

I confirm that I have read this submission and believe that I have an appropriate level of expertise to confirm that it is of an acceptable scientific standard.

# Response

**Published:** 2022-02-04

**Authors:** Yuanyuan Zhou

**Affiliations:** Baylor College of Medicine, USA

Thank you for your time to review this paper and provide feedback.
